# Genomic characterization of rabies virus glycoprotein co-expressing CD70 CAR-T cells during killing of glioma cells *in vitro*

**DOI:** 10.3389/fimmu.2026.1680513

**Published:** 2026-04-06

**Authors:** Feng Ji, Kexing Gao, Xianchen Wu, Hao Lin

**Affiliations:** 1 Office of Research Platform Management, Zhongda Hospital, School of Medicine, Southeast University, Nanjing, China; 2 National Medical Research of Industry-Education Integration Innovation Platform for Interventional Medica Engineering, Zhongda Hospital, Southeast University, Nanjing, China; 3 Department of General Surgery, Shuyang Zhongxing Hospital, Suqian, China

**Keywords:** blood–brain barrier, CAR-T, glioma, immunotherapy, RVG29

## Abstract

**Background:**

Chimeric antigen receptor T cell therapy has limited efficacy in the treatment of glioma due to the blood-brain barrier and T cell exhaustion. Enhancement with rabies virus glycoprotein (RVG29) has shown improved brain-related efficacy.

**Methods:**

In this study, we developed CD70R CAR-T cells by modifying anti-CD70 CAR-T cells with RVG29 and tested their tumor-killing ability *in vitro*.

**Results:**

Transcriptomic profiling via RNA-seq revealed the activated signaling pathways in modified CD70R CAR-T cells. These cells displayed effective *in vitro* cytotoxicity against CD70^+^; glioma cells, furthermore, co-culture with glioma cells promoted the expansion of quiescent CD70R CAR-T cells.

**Conclusion:**

The RVG29 modification enhances CAR-T therapy for brain tumors and holds promise for treating glioma.

## Highlights

- Modification of RVG leads to differences in activated signaling pathways.
- CD70R CAR-T cells exhibit enhanced antitumor potential against glioma cells *in vitro*.
- CD70R CAR-T showed long-term killing potential.

## Introduction

Gliomas are the most common primary malignant brain tumors, accounting for approximately 81% of all central nervous system (CNS) malignant tumors (1, 2). Glioblastoma (GBM), the most aggressive diffuse glioma (WHO grade IV), is also by far the most common (3, 4). Due to its highly proliferative and infiltrative nature, GBM has a median survival rate of only about 14.6 months, with fewer than 5% of patients surviving for five years (5). Chimeric antigen receptor T cell (CAR-T) therapy is an emerging immunotherapy with encouraging clinical responses in hematological malignancy (6). It is now being explored for treating various solid tumors, including GBM (7–9). However, there are several challenges hinder the implementation of CAR-T therapy in solid tumors, such as identifying homogeneously expressed tumor-associated or tumor-specific target antigens, enabling the infiltration of CAR-T cells into tumor tissue, and ensuring that the immunosuppressive microenvironment does not interfere with the function and persistence of CAR-T cells (10, 11). In addition, most brain tumors are located behind the blood-brain barrier (BBB), which limits access to the tumor if treatment is administered orally or intravenously (12–14).

Targeted drug delivery and imaging for brain diseases such as tumors, Alzheimer’s disease, and Parkinson’s disease have been extensively studied over the years. The delivery of therapeutic protein, such as enzymatic toxin, to the diseased brain is of great interest because a small amount of protein can effectively cure the disease (15). The BBB controls the passage of substances from the blood into the CNS and protects against foreign organisms and noxious chemicals with a very strong membrane system (16, 17). Physiologically, the BBB consists of an endothelial cell layer, which forms the walls of capillaries and contains tight junctions (18). Extensive evidence shows that all gliomas exhibit clinically significant tumor burden with an intact BBB, making drugs with poor BBB permeability ineffective against these tumors (19). Traditional methods of temporarily opening the BBB pose a risk of brain damage, especially for patients who have relapsed after surgery. Consequently, intracranial injection of CAR-T cells is no longer recommended in these conditions.

Brain-targeted nanocarriers, particularly polymeric nanoparticles, have recently emerged as a promising solution for drug delivery across the BBB. The polypeptide rabies virus glycoprotein (RVG) 29 can specifically bind to the nicotinic acetylcholine receptor (AchR) at the BBB and facilitate its crossing (18, 20, 21). Conjugation with RVG29 enhances the brain-related efficacy of various agents, including nucleic acids, proteins, and nanoparticles, when administered systemically (22, 23). We have explored the positive therapeutic effects of RVG29-mediated CAR-T cell delivery in intracerebral applications (24). However, its further application potential remains unexplored.

In this study, we developed CD70R CAR-T, which exhibited enhanced cytotoxicity against CD70-expressing glioma cells *in vitro*, accompanied by accelerated proliferative capacity and reduced expression of exhaustion-related markers. Collectively, these findings highlight the promising potential of CD70R CAR-T cells as a targeted immunotherapeutic strategy for GBM.

## Materials and methods

### Cell lines and patient tissue samples

Peripheral blood mononuclear cells (TPCS#PB025C) were purchased from Miles-Bio (Shanghai, China). All specimens were collected in accordance with protocols approved by the Nanjing First Hospital institutional review board, and written informed consent was obtained from each donor. Human glioma cell line U251 and the HEK293 cell line were obtained from the American Type Culture Collection. All cell lines were tested and authenticated by short tandem repeat profiling (DNA fingerprinting) within 6 months of the study and routinely tested for mycoplasma species before all experiments. All cells grew in the recommended medium supplemented with 10% Fetal Bovine Serum (FBS) in a 10% CO_2_ incubator. T lymphocytes were maintained in X-VIVO 15 serum-free hematopoietic cell medium supplemented with 5% FBS 2 ng/mL human recombinant interleukin (IL)-2 (Sigma, Germany).

The acquisition of tumor samples in this study was approved by the Ethics Committee of Nanjing First Hospital (201806-AP-1), in accordance with the ethical principles of Good Clinical Practice and the Declaration of Helsinki. Written informed consent was obtained from all patients.

### Flow cytometry

For flow cytometry analysis of human cells and glioma cell lines, cells were stained with mouse anti-human CD70-APC (Abcam, Grand Island, NY, USA) and mouse anti-human CD3-FITC, goat anti-human IgG-Fc gamma fragment specific-APC, mouse anti-human CD4-PE, mouse anti-human CD8-APC, Isotype Control Antibody, rat IgG2b, APC (all from BD Biosciences) monoclonal antibodies diluted at 1:100. Expression of CAR proteins was detected with biotinylated recombinant APC-Labeled Human CD27 Ligand/CD70 Protein (Sigma-Aldrich Co., USA). Monoclonal Anti-6X His IgG (H+L), CF™ 594 antibody (Thermo fisher, USA). Cells were stained with antibodies for 30 min at room temperature. All samples were acquired on a CytoFLEX S (Beckman Coulter, Indianapolis, IN, USA), and data were analyzed using Kaluza 2.1 (Beckman Coulter Life Sciences).

### Retroviral vector construction

The CD70 CAR (CD8α signal peptide-anti CD70 scFv-CD8α Hinge-CD8α™-4-1BB-CD3ζ) was designed and completely synthesized in our previous study based on the structure of the second-generation CAR. We linked the CD70 CAR to the RVG29 protein via the T2A peptide and codon optimized the complete CAR sequence using GenSmart™ technology. These plasmids were linearized with EcoR I/BamH I and subcloned into a pMDL-based expression vector that contained the green fluorescent protein gene as a tracker to detect the expression of CAR with FACS.

### Lentivirus production

The lentiviral packaging scheme followed the same approach as in our previous study (25). Concentrated viral stocks were prepared by centrifuging the viral supernatant in an SW28 rotor at 25,000 × *g* at 4 °C for 2 h. The supernatant was decanted, and 200 µL TNE (50 mM Tris, pH 7.8; 30 mM NaCl, 1 mM EDTA, pH 8.0) was added. After sitting overnight at 4 °C, the virus was resuspended and stored at –80 °C.

### T cell isolation and lentivirus transduction

T cells were isolated from peripheral blood mononuclear cells (Miles-Bio, Shanghai) by negative selection using the EasySep™ human T cell isolation kit (Stemcell, Canada). Primary lymphocytes were stimulated with anti-CD3/CD28 Dynabeads (Stemcell, Canada). Detection of CAR-T cell positive rate and detection of cell phenotype by lentivirus infection and continuous culture for 48 h after T cell isolation. Cells were allowed to expand in culture until day 15. For all experiments using CD70R CAR-T cells, paired (from the same donor) non-transduced T cells, activated and cultured for an equivalent time, served as control T cells, CD70 CAR-T, or Mock CAR-T.

### Generation of CAR-modified T cells and gene-modified cell lines

The transduction of CAR genes for the preparation of CAR-T cells was performed as mentioned in our previous study (24). Transduction efficiency was measured by flow cytometry 5–7 days after transduction. To track the number of T cells over time, we manually counted viable cells using trypan blue. Although T cells were generated in X-VIVO 15 medium, all *in vitro* functional assays were performed in mixed medium (50% DMEM + 50% X-VIVO 15) supplemented with 10% FBS.

### Impedance-based kinetics cell lysis assay

Tumor cell lysis kinetics were assessed via a real-time cell analyzer (RTCA, ACEA, San Diego, CA) over 50 hours. Human glioma cells were seeded at 2.0 × 10^4^ cells/well in 96-well impedance plates (three replicates per group). After 24 hours of culture, effector cells (T cells, 70R CAR-T, CD70R CAR-T) were added at an E:T ratio of 1:1, and impedance was measured every 15 minutes. After 24 h, effector T cells were added to the unit at various ratios of effector cells (T cells, 70R CAR-T, CD70R CAR-T) to target cells. Impedance was measured in 15 min intervals. The impedance-based cell index for each well and time point was normalized to the cell index prior to the addition of the T cells. The kinetics of tumor cell lysis is depicted as the change in the normalized cell index over time. Cell culture supernatants were harvested and assayed for IL-2, tumor necrosis factor (TNF)-α, and interferon (IFN)-γ 6 h later.

### Quantification of cytokines

Human IL-2, TNF-α, and IFN-γ were assessed on the culture supernatant collected during the cell killing procedure described above using the R&D ELISA development kit according to the manufacturer’s protocol. In mice studies collected blood was left at room temperature for 20 minutes to clot, then centrifuged to obtain serum. Levels of IL-2, TNF-α, and IFN-γ were detected with ELISA kits according to standard manuals. Each experiment was performed independently 3 times.

### RNA sequencing and data analysis

70R CAR-T and CD70R CAR-T cells were co-cultured with U251 cells, followed by separation and RNA extraction. Three biological replicates were performed, and the RNA from each replicate was independently sequenced. Specifically, CAR-T cells were incubated with U251 cells at a 2:1 ratio for 6 hours. The suspended CAR-T cells were then collected, separated from the adherent tumor cells, and centrifuged at 500 × g for 4 minutes. Prior to RNA extraction and sequencing, dead CAR-T cells were removed using a Dead Cell Removal Kit (Miltenyi Biotec, USA). The RNA products from the three replicates of CAR-T cells were sent for RNA sequencing (Wekemo Bioinformatics). For data analysis, the paired-end reads were mapped to the human genome using TopHat with default parameters (http://tophat.cbcb.umd.edu) and assembled using the Cufflinks pipeline (http://cufflinks.cbcb.umd.edu/). Gene expression was quantified by RNA-seq using the Illumina HiSeq v2 platform.

### Immunofluorescence/immunohistochemistry

Tumor cell surface expression of CD70 was detected with CD70 scFv.mFc chimeric proteins followed by Cy3-conjugated goat anti-mouse Fc (Proteintech) for CD70 scFv. hFc. Cells were blocked with 5% bovine serum albumin and incubated with chimeric proteins for 2 h at 4 °C. For secondary staining, cells were washed three times and incubated with a secondary antibody in the dark for 60 minutes at 4 °C. Images were captured using a confocal microscope. Tumor tissue was harvested and fixed in 4% paraformaldehyde (Boston BioProducts) for 48 to 72 h, then stored in 70% ethanol until further processing. Paraffin-embedded tumor sections (10 µm) were deparaffinized followed by heat-mediated antigen retrieval for 30 min in IHC-Tek epitope retrieval solution (IHC World). After antigen retrieval, tumor sections were permeabilized with 100% methanol. Sections were blocked for 30 min with Tris-NaCl (TNB) blocking buffer (PerkinElmer) and then incubated with rabbit anti-vaccinia virus antibody (Abcam) diluted 1:100 in TNB blocking buffer overnight in a humidified chamber at 4 °C. After incubation, tumor sections were washed and incubated with Alexa Fluor 488-conjugated goat anti-rabbit secondary antibody (Abcam) for 1 h at room temperature. Nuclei were counterstained with DAPI. Deparaffinized tumor sections (10 µm) were stained with mouse anti-human CD3 (Abcam). Images were obtained with a 3DHISTECH Pannoramic digital slide scanner and the associated CaseViewer software (3DHISTECH). For CD3 quantification after IHC staining, ImageJ (NIH) analysis was performed according to the standard recommended algorithm (26–28).

### RNA isolation and real-time PCR

RNA was isolated using the RNeasy Mini Kit (Qiagen, Hilden, Germany) and subsequently reverse-transcribed into complementary DNA (cDNA) using the High-Capacity cDNA Reverse Transcription Kit (Takara, Dalian, China) according to the manufacturers’ instructions. Quantitative real-time polymerase chain reaction (qRT-PCR) was performed on a qTower Real-Time PCR System (Analytik Jena, Jena, Germany) with the SYBR Green Master Mix (Vazyme, Nanjing, China) using the primers listed in **Supplementary Table 1**.

### TaqMan probe-based quantitative PCR analysis of CD70R CAR-T cell DNA copy number

Real-time quantitative polymerase chain reaction (qPCR) was performed to quantify the DNA copy number of CD70R CAR-T cells in genomic DNA samples isolated from *in vitro* cytotoxicity assay cultures. Briefly, T cells were harvested following completion of the *in vitro* cytotoxicity assays, and genomic DNA was subsequently extracted. For qPCR detection, the forward primer (5′-GAGGAACAGTGACCCTGACA-3′), reverse primer (5′-CGCCGGAGTGTCTTGTATTG-3′) were utilized, and probe primer 5’6-FAM- TGTCGCTGGTCACGCTGCCA-TAMRA-3’6 was used in the qRT-PCR assay.

### Human immunome

Human immune-related genes were obtained from the ImmPort project (http://www.immport.org) (29, 30). A total of 1811 genes in 17 immune-related pathways were obtained. In addition, we also obtained genes that are identified as essential for immune response from recent literature (31). These genes were defined as essential immunotherapy genes and included in the 18th pathway. In total, 2,273 immune-related genes were obtained.

### Statistical analysis

For the screening assay, statistical significance was evaluated using an unpaired, two-tailed t-test. For the comparison of 3 or more groups with 2 or more independent variables, statistical significance was determined by multiple t-tests or two-way ANOVA with Sidak’s or Tukey’s multiple comparisons test. Statistical significance between survival curves was determined using the log-rank (Mantel-Cox) test. Bioluminescence imaging data were analyzed using either ANOVA or the t-test. P values were calculated using GraphPad Prism 8.0 software. Throughout the study ns: no significance; *P < 0.05; **P < 0.01; ***P < 0.001, ****P < 0.0001.

## Result

### RVG29 and CD70 CAR are co-expressed to modify T cells to generate CD70R CAR-T

**Figure 1:**
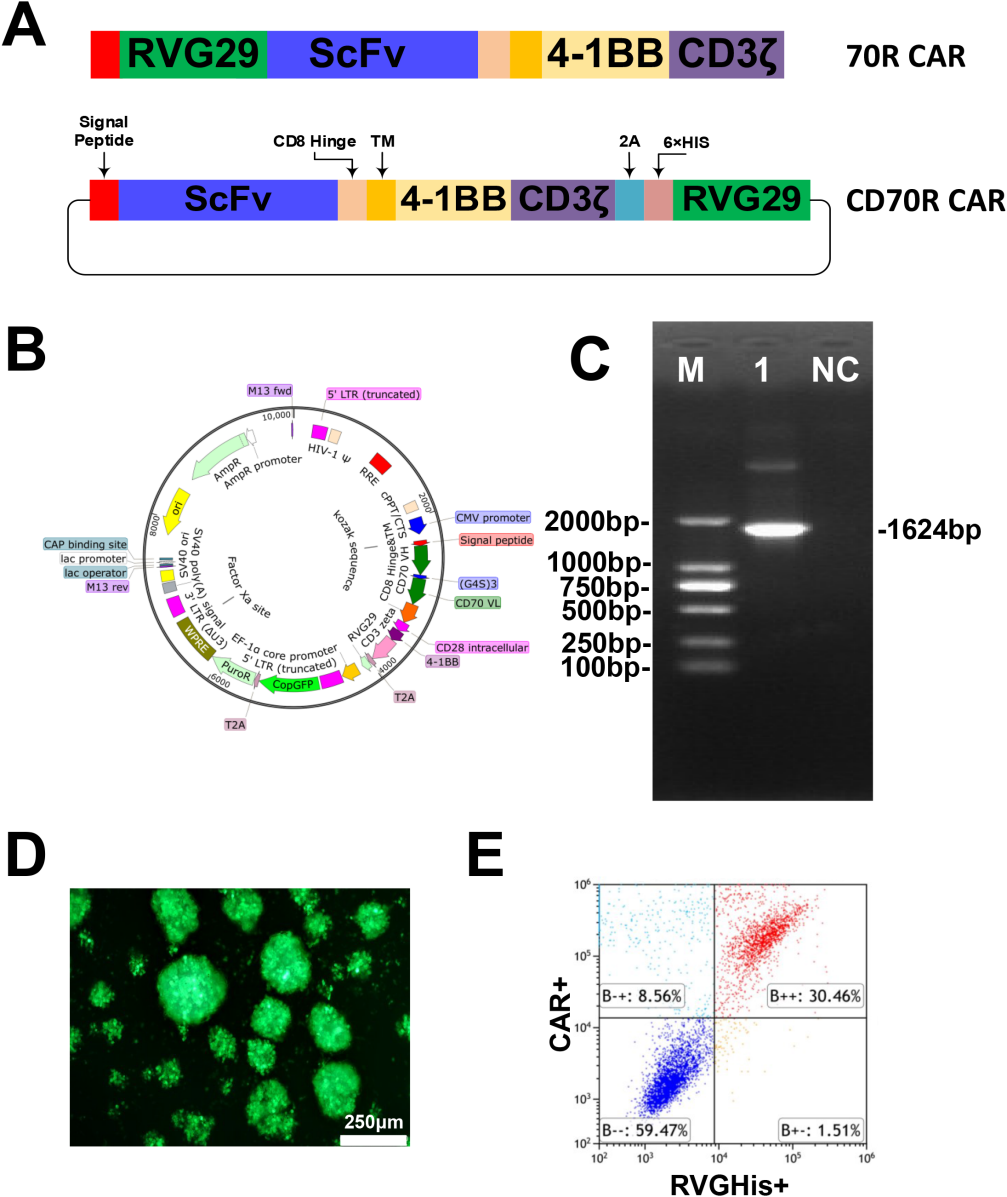
Construction of RVG29-modified CAR-T cells. **(A)** CD70R CAR: Schematic of CD70R CAR containing different spacer regions [CD8α signaling peptide, IgG4 hinge, (G4S)_3_ connector and CD8α™] and co-stimulatory structural domains (CD28); 70R CAR: The design in the previous study. **(B)** The lentiviral backbone plasmid encodes the CD70R CAR. **(C)** PCR validation of E. coli encoding the retroviral vector of CD70R CAR. **(D)** Microscopic image of CD70R CAR-T cells expressing a GFP luciferase reporter gene on day 10 of transfection (Scale bar = 250 µm). **(E)** Representative dot plots of flow cytometry CAR expression analysis at day 10 after transfection of T cells.

The CARs were composed of a second-generation architecture containing the CD8α hinge and transmembrane domain along with the 4-1BB and CD3ζ signaling domains. The anti-CD70-specific scFv was derived from our previous study (24, 25). VL and VH regions of the antigen-binding domains were connected via a (G_4_S)_3_-linker. To ensure efficient separation during transgene translation, a T2A sequence was constructed between the CAR and RVG29 (**Figure 1A**). This study employed a lentiviral gene modification system to establish a production scheme for CAR-T cells, constructing CD70R CAR. The CD70R CAR was inserted into the pMDL lentiviral vector using double enzyme digestion with EcoR I and BamH I (**Figure 1B**). The results of double enzyme digestion confirmed that the target gene was successfully ligated into the pMDL vector (**Figure 1C**). After packaging the lentivirus according to the reference protocol, T cells isolated from healthy donor peripheral blood mononuclear cells (PBMCs, TPCS#PB025C) were infected with the lentivirus carrying the CD70R CAR to construct CD70R CAR-T cells. Microscopic observation revealed clumping growth of the cells, indicating rapid proliferation and GFP expression suggesting a high rate of CAR positivity (**Figure 1D**). Flow cytometry analysis for CAR/RVG expression showed a high positivity rate of up to 30.46% (**Figure 1E**, **Supplementary Figure S1**).

### Validation of CD70R CAR-T *in vitro* killing function

**Figure 2:**
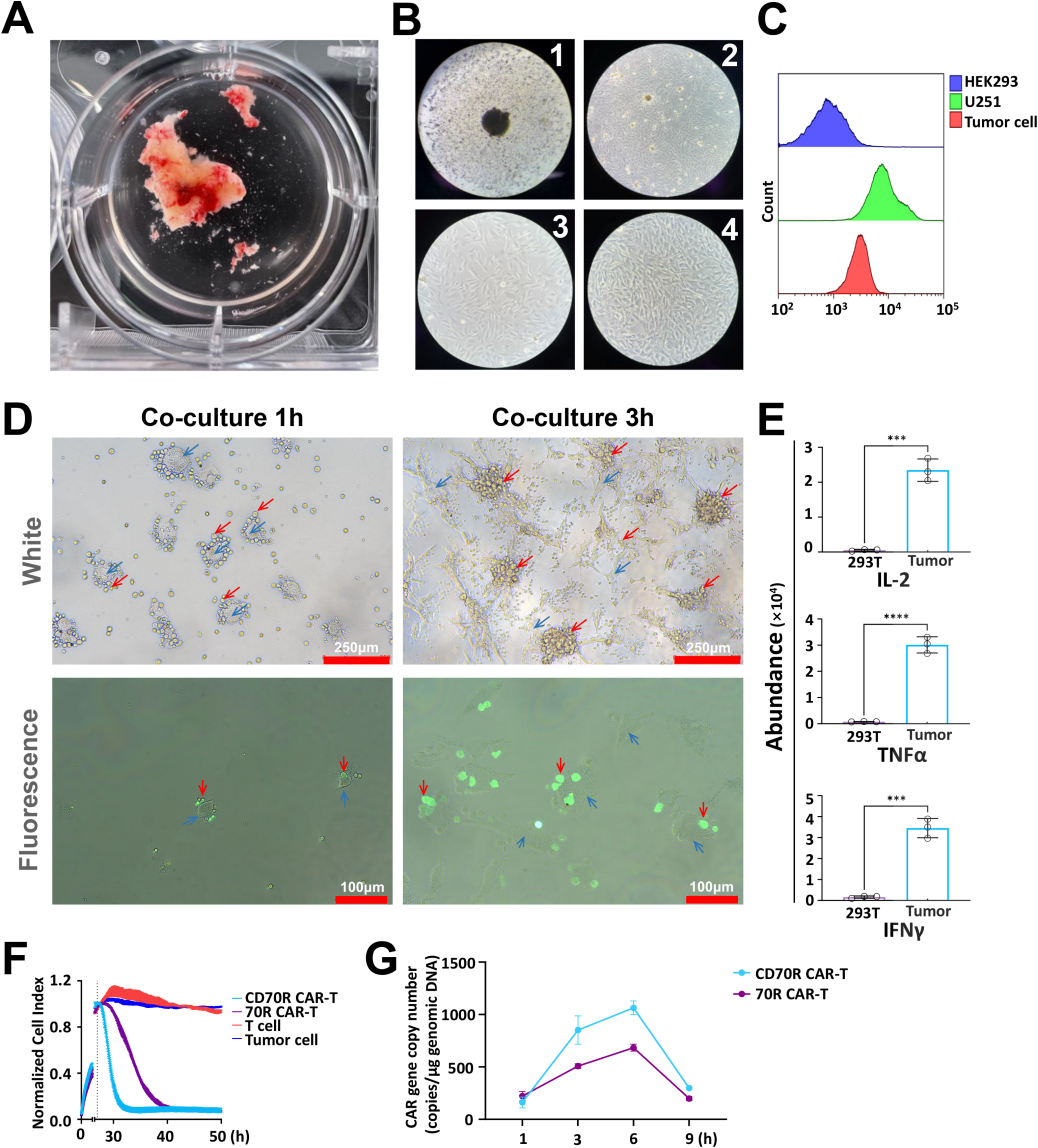
CD70R CAR-T cells recognize and kill patient-derived glioma cells. **(A)** Isolated tumor tissue samples from glioma patients. **(B)** Culture of patient-derived glioma tissue samples. **(C)** Flow cytometry analysis was conducted to detect CD70 antigen expression in target cells (U251, patient-derived glioma cells) and HEK293 cells. **(D)** Microscopic observations of patient-derived glioma cells and CD70R CAR-T cells co-cultured for 1 and 3 hours (Red arrows indicate CAR-T cells, blue arrows indicate tumor cells). **(E)** ELISA results show IL-2, TNF-α, and IFN-γ secretion levels for CD70R CAR-T cells encountering patient-derived glioma cells and 293T (MSLN⁻) cells for 6h. Data are mean ± SD, n = 3. **(F)** *In vitro* expansion capacity of multi-group CAR-T cells. Data are mean ± SD, n = 3. **(G)** qPCR quantification of CAR copy number (targeting the WPRE region) was conducted at 1, 3, 6, and 9 h post-co-culture. Data are mean ± SD, n = 3. ns: no significance; ***P < 0.001; ****P < 0.0001.

After excising the patient’s tumor tissue, it was inoculated into a 6-well plate supplemented with Phosphate-Buffered Saline (PBS) (**Figure 2A**). Under sterile conditions, the tissue was cut into blocks of approximately 1 mm³ in size, rinsed several times with cold PBS to remove small fragments and erythrocytes, and the PBS was then discarded. Subsequently, the tissue was supplemented with RPMI-1640 medium containing 10% Fetal Bovine Serum (FBS) and placed in a CO_2_ incubator for culture. As the culture progressed, cells from these tissue blocks gradually detached and adhered to the wall, proliferating over time into a mixed cell population that resembled a cell line (**Figure 2B**). Flow cytometric analysis of target antigen expression revealed that both the glioblastoma cell line U251 and patient-derived glioblastoma cells exhibited significantly high levels of this antigen compared with human embryonic kidney 293 (HEK293) cells (**Figure 2C**). The CAR-T cells were co-cultured with the patient’s tumor cells for 1 and 3 hours, allowing the process of CAR-T cells recognizing and killing the tumor cells to be observed under a microscope (**Figure 2D**). Note that cytokines (IL-2, TNF-α, and IFN-γ) released by CD70R CAR-T cells were only observed on co-culture with the patient-derived glioma cells at the E: T = 2:1 ratio, which highlights the antigen specificity of the target cell lysis (**Figure 2E**). Real-time cell analysis (RTCA) demonstrated that all CAR-T cells effectively lysed glioma cells at the E:T ratio of 2:1. Among these, CD70R CAR-T cells exhibited the most rapid tumor cell killing kinetics (**Figure 2F**). Pharmacokinetic evaluation via real-time quantitative PCR (qPCR) was performed to quantitatively assess CAR-T cell expansion and persistence *in vitro*. After co-cultivation at an E:T ratio of 1:1 for 1, 3, 6, and 12 hours, the peak CAR gene copy number reached 1,115.27 copies/μg genomic DNA (**Figure 2G**).

### Transcriptome sequencing reveals differences in the CAR-T cell killing process before and after optimization

**Figure 3:**
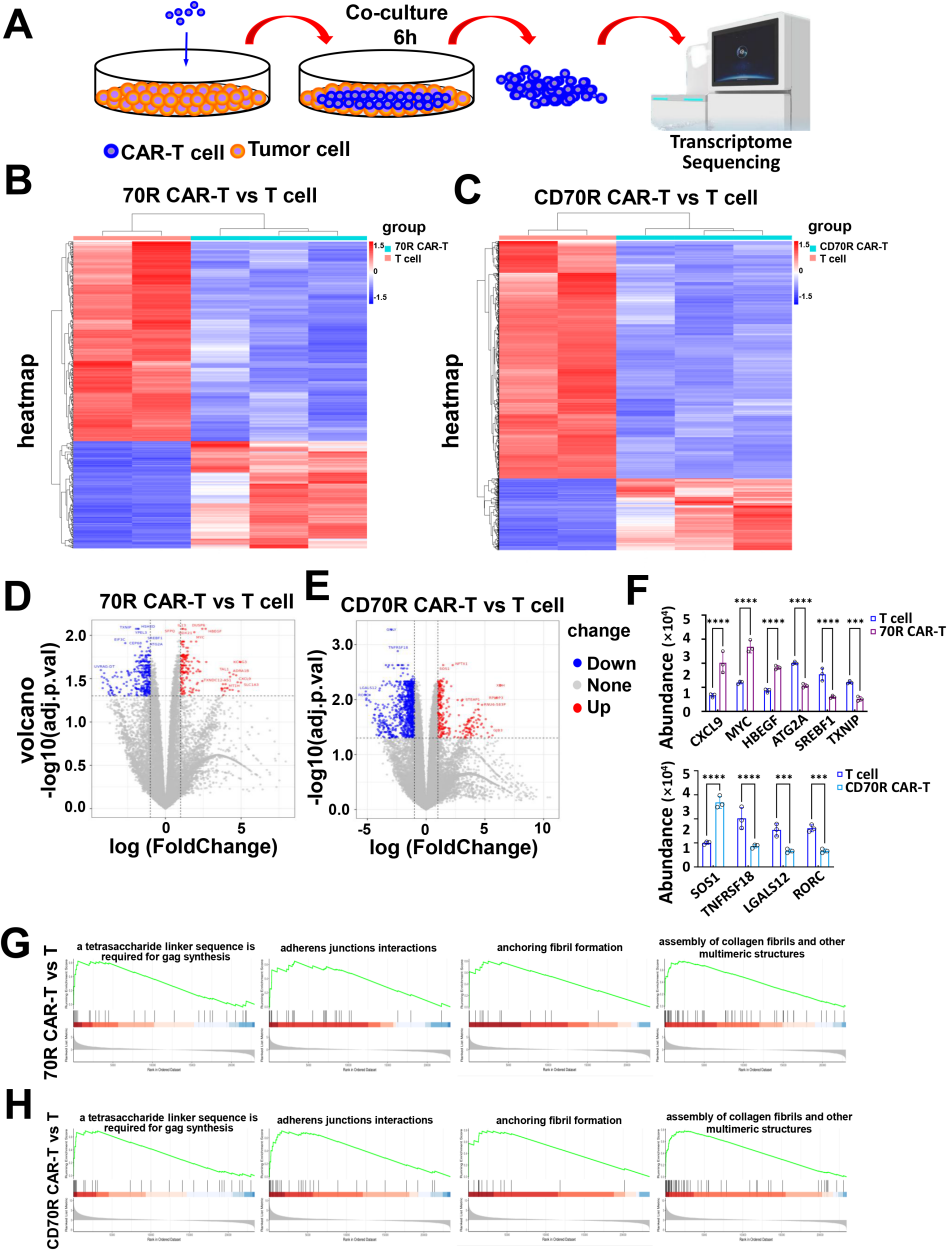
Effect of RVG29 modification on CAR-T cell signaling. **(A)** Schematic diagram of CAR-T cell stimulation and RNAseq sample preparation. U251 cells were co-cultured with 70R CAR-T, CD70R CAR-T cells respectively for 6 h. CAR-T cells were isolated and collected for RNAseq sequencing. RNAseq analysis of 70R CAR-T **(B)** and CD70R CAR-T **(C)** cells under U251 cell stimulation conditions. 70R CAR-T **(D)** and CD70R CAR-T **(E)** cells were co-cultured with U251 cells for 6 hours and then sorted out for transcriptome sequencing, and the volcano plots show fold changes in gene expression (compared with T cells, respectively). **(F)** Following 6 h of co-culture between CAR-T cells and tumor cells, we assessed the expression levels of the target genes by qPCR. Data are mean ± SD, n = 3. **(G)** U251 cell stimulation conditions 70R CAR-T cells under U251 cell stimulation conditions (GSEA). **(H)** GSEA of CD70R CAR-T cells under U251 cell stimulation conditions. ns: no significance; ***P < 0.001; ****P < 0.0001.

To investigate how RVG29 modification enhances the anti-tumor activity and durability of CD70 CAR-T cells, we performed RNA sequencing (RNAseq) analysis. Following co-culture with the glioma cell line U251 for 6 hours, CAR-T cells were isolated for sequencing (**Figure 3A**). RNAseq quality control results are shown in **Supplementary Figures** (**Supplementary Figures S2**-**S11**). We observed distinct gene expression patterns in both 70R CAR-T and CD70R CAR-T cells compared to unmodified T cells (**Figures 3B, C**). In comparison with T cells, 70R CAR-T cells showed significant upregulation of genes associated with MYC, HBEGF (log2FC: 1.00, p=0.05), while genes related to SREBF1, ATG2A, TXNIP were significantly downregulated (log2FC: -1.00, p = 0.05). In CD70R CAR-T cells, there was significant upregulation of genes linked to SOS1whereas genes related to LGALS12, RORC, TNFRSF18 were significantly downregulated (log2FC: -1.00, p = 0.05). Gene expression of CD70R CAR-T cell products during killing differed from that of 70R CAR-T. Similar to the phenotypic results described above, most memory-related genes, such as SOS1, which was highly expressed in 70R CAR-T cells, included MYC and CXCL9 (log2FC: 1.00, p=0.05), furthermore, the CD70R CAR-T cell the expression of activation- and exhaustion-related genes, such as TNFRSF18, LGALS12, was significantly downregulated (log2FC: -1.00, p=0.05) (**Figures 3D, E**). We verified the expression levels of the relevant genes by qPCR, confirming consistency with our RNA-seq results (**Figure 3F**). Gene Set Enrichment Analysis (GSEA) revealed that in CD70R CAR-T cells, there was an increase in glycolysis pathways and interferon (IFN) signaling (**Figures 3G, H**).

### Study on T cell activation and metabolic adaptability of CD70R CAR-T cells

**Figure 4:**
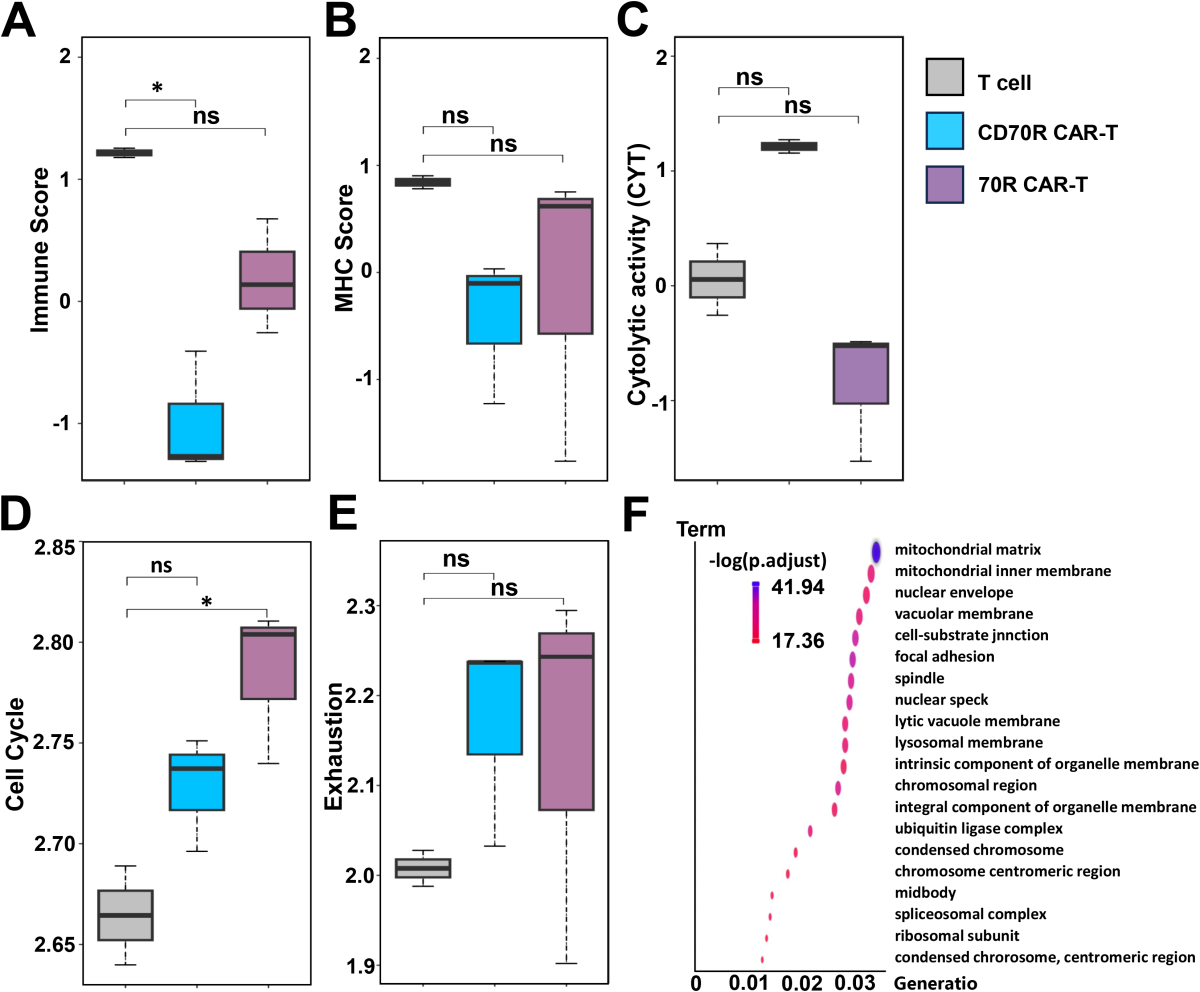
Differential changes in T cell function after 6 hours of co-culture of CAR-T cells and U251 cell line. T cells were isolated for transcriptome sequencing using mock T, 70R CAR-T or CD70R CAR-T cells co-cultured with U251 cell line for 6 h. Immune Score **(A)**, MHC Score **(B)**, Cytolytic activity (CYT) **(C)**, Cell Cycle **(D)** and Exhaustion **(E)**. Data are mean ± SD, n = 3. **(F)** Co-culture with U251 cells for 6 hours, and compare the KEGG enrichment analysis of the differentially expressed genes (DEGs) in CD70R CAR-T with that of the differentially expressed genes in CD70R CAR-T. ns: no significance; *P < 0.05.

The Immune Score, used as an indicator to assess immune response against tumors, showed that CD70R CAR-T cells infiltration was weaker than 70R CAR-T cells (**Figure 4A**). However, the MHC Score comparison results showed that the antigen presentation ability of the tumor cells was not affected (**Figure 4B**). Cytolytic activity results demonstrated that CD70R CAR-T cells have a stronger killing capacity against tumor cells than 70R CAR-T cells (**Figure 4C**). Cell cycle comparison results indicated that CD70R CAR-T cells have a shorter cell cycle compared to 70R CAR-T cells after stimulation by tumor cells (**Figure 4D**), suggesting that CD70R CAR-T cells can proliferate more rapidly. Analysis of the immune cell exhaustion state showed that CD70R CAR-T cells have a similar level of exhaustion to 70R CAR-T cells after stimulation by tumor cells (**Figure 4E**). T cells were collected after CD70R CAR-T killing of the U251 glioma cell line, and RNAseq analysis to detect the enrichment of functionally related genes GO BP and KEGG pathways showed that cell function-related features are mainly affected by organelle or cell structure changes in pathways such as mitochondrial matrix, mitochondrial endochondral membrane, nuclear membrane, vesicle membrane, cell-matrix junctions, focal adhesions, fusiform bodies, nuclear speckles, lysosomal vesicle membranes, lysosomal membranes, intrinsic components of organelle membranes, and chromosomal regions (**Figure 4F**).

### Co-expression of RVG29 leads to differences in the cytotoxic killing activity of CAR-T cells *in vitro*

**Figure 5:**
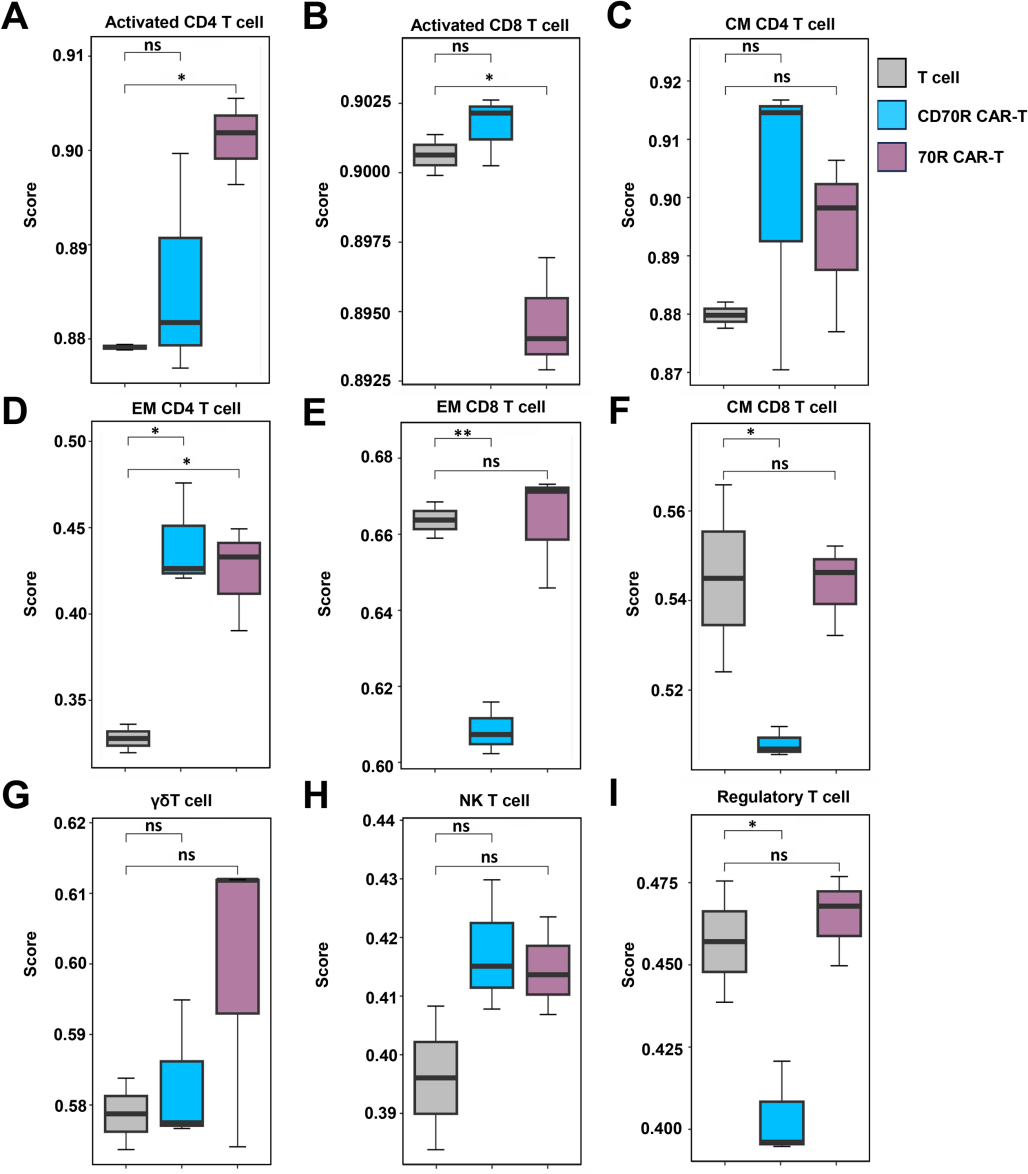
Differences in T cell phenotypic changes after 6 hours of co-culture of CAR-T cells and U251 cell line. T cells were isolated for transcriptome sequencing using mock T cells, 70R CAR-T cells, or CD70R CAR-T cells co-cultured with the U251 cell line for 6 h. Activated CD4 T cells **(A)**, Activated CD8 T cells **(B)**, CM CD4 T cells **(C)**, EM CD4 T cells **(D)** EM CD8 T cell **(E)**, CM CD8 T cell **(F)**, γδ T cell **(G)**, NK T cell **(H)**, Regulatory T cell **(I)**. Data are mean ± SD, n = 3. ns: no significance; *P < 0.05; **P < 0.01.

T cells are a type of immune cell characterized by a variety of surface markers and functional molecules. RNAseq analysis was performed on CD70R CAR-T cells and 70R CAR-T cells after co-culturing with tumor cells for 6 hours. The results showed significant differences in the activation of different T cell subsets. Compared to T cells, there was a significant increase in Activated CD4 T cells in 70R CAR-T cells, while no significant change in activated CD4 T cells was observed in CD70R CAR-T cells (**Figure 5A**). Activated CD8 T cells were significantly reduced in 70R CAR-T cells, with no significant change in CD70R CAR-T cells (**Figure 5B**). Central memory (CM) CD4 T cells showed no significant changes in both CD70R CAR-T cells and 70R CAR-T cells (**Figure 5C**), whereas effector memory (EM) CD4 T cells were significantly increased in both (**Figure 5D**). EM CD8 T cells were significantly decreased in CD70R CAR-T cells and showed almost no change in 70R CAR-T cells (**Figure 5E**). CM CD8 T cells showed no significant differences in either CD70R CAR-T cells or 70R CAR-T cells (**Figure 5F**). Although slight changes were observed in γδ T cells, NK T cells, and regulatory T cells after co-culture with tumor cells for both CD70R CAR-T cells and 70R CAR-T cells, these differences were not significant (**Figures 5G-I**).

## Discussion

RVG29, a 29-residue peptide derived from the rabies virus glycoprotein (RVG), has enabled the delivery of multiple drugs to the central nervous system (CNS) for treating neurological disorders and has demonstrated a favorable safety profile (32–34). CD70, a member of the tumor necrosis factor receptor (TNFR) superfamily, is highly expressed in glioblastoma (GBM) and associated with poor prognosis, making it a promising target for cellular immunotherapy in GBM (35). In 2023, we first innovatively utilized RVG29 to modify CD70-CAR-T cells, enhancing their efficacy following intravenous administration for GBM treatment (24). In the present study, we optimized the RVG-modified CD70 CAR-T system by altering RVG29 from its original coupling with CAR (70R CAR) to co-expression with CAR on the T cell surface (CD70R CAR), aiming to improve its potential for clinical translation.

The BBB comprises endothelial cells, basement membranes, and astrocytic foot processes that surround cerebral blood vessels (36). It acts as a physiological barrier protecting the central nervous system (CNS) by separating it from the peripheral circulation, which contains inflammatory mediators and immune cells (37). The BBB plays a critical role in maintaining the stability of the brain microenvironment by tightly regulating the entry of specific nutrients from the bloodstream into the brain while preventing the passage of harmful substances and pathogens (38). Owing to the BBB’s protective properties, most drugs encounter significant challenges in achieving therapeutic concentrations within the brain. For instance, a study demonstrated that intravenously administered immunoglobulins were only minimally detectable in the brains of C57BL/6 mice, with levels accounting for a mere 0.009 ± 0.001% of those measured in plasma (39). This underscores the formidable barrier that the BBB poses to pharmacological interventions for CNS disorders.

To address these challenges, researchers have explored various strategies to enhance drug penetration across the BBB, including receptor-mediated transcytosis (RMT), neuroviral vectors, and nanoparticle-based delivery systems (38). For instance, RVG29 employs RMT to traverse the BBB by specifically binding to neuronal cells expressing the nicotinic acetylcholine receptor (nAchR), thereby facilitating targeted delivery to the brain. This peptide has been applied in brain-targeted gene delivery systems (22), modified nanocarriers for precise drug delivery (40, 41), and cellular engineering (42). While these advancements hold promise for the treatment of CNS-associated tumors, they also pose challenges related to safety and efficacy.

In this study, we demonstrated via *in vitro* cytotoxicity assays that although the Immune Score of CD70R CAR-T cells was lower than that of 70R CAR-T cells, their cytolytic activity was enhanced. Additionally, cell cycle analysis revealed that CD70R CAR-T cells exhibited a shorter cell cycle, indicating they can replicate more rapidly to generate tumor-killing immune cells within the same timeframe. During the cytotoxic process, 70R CAR-T cells showed a significant increase and decrease in the levels of activated CD4^+^; and CD8^+^; T cells, respectively, compared to unmodified T cells. In contrast, the levels of activated CD4^+^; and CD8^+^; T cells in CD70R CAR-T cells remained nearly unchanged. This finding suggests that CD70R CAR-T cells possess more durable tumor-killing capacity, maintaining their functionality and persistence throughout the anti-tumor response.

Intracranial injections can induce brain parenchymal injury, which exacerbates blood-brain barrier (BBB) disruption, leading to increased permeability, inflammatory responses, and elevated intracellular reactive oxygen species (ROS) levels (43). RVG peptides are commonly expressed on the surface of nanoparticles for brain targeting following intravenous administration (44–46). Thus, the intravenous route is preferred to avoid these complications. Given the inherent limitations of the BBB, optimizing drug design and developing novel strategies to facilitate BBB traversal are of particular importance (47).

Despite the plethora of CAR-T therapies currently in clinical trials, none have been approved for the treatment of solid tumors. The complex immunosuppressive tumor microenvironment, dense stroma, abnormal vascular architecture, and tumor antigen heterogeneity of solid tumors pose substantial challenges to CAR-T cell therapy (48). For targeted therapy research in GBM, existing targets include EGFRvIII, IL13Rα2 (49), GD2 (50), CD70 (51), and HER2 (52), which are highly expressed in GBM but minimally expressed in normal brain tissue. However, no significant success has been achieved in clinical trials, partly due to the presence of the BBB, which restricts the infiltration of CAR-T cells into the tumor parenchyma (53). Achieving efficient and safe BBB crossing for CAR-T cell therapy targeting glioma remains a key focus of our future research.

In the present study, we optimized RVG29-modified CD70 CAR-T cells and confirmed via *in vitro* cytotoxicity assays that these cells can efficiently eliminate patient-derived glioma cells. Additionally, we demonstrated that the optimized CD70R CAR-T cells exhibit enhanced expansion compared to the previous design, which supports a more sustained anti-tumor effect *in vivo*. This optimization establishes RVG29-modified CD70 CAR-T cells as a promising therapeutic strategy for GBM treatment.

## Data Availability

The data presented in the study are deposited in the Genome Sequence Archive (GSA) of National Genomics Data Center repository, accession number HRA016320.
