# Supplementary material for: Genomic characterization of rabies virus glycoprotein co-expressing CD70 CAR-T cells during killing of glioma cells in vitro
Source: Front Immunol. 2026 Apr 6;17:1680513. doi: 10.3389/fimmu.2026.1680513 (PMC13093881; doi:10.3389/fimmu.2026.1680513)
Supplement: Supplementary file 1 [file Supplementaryfile1.docx]

**Supplementary Figures and Table**

**Genomic characterization of rabies virus glycoprotein co-expressing CD70 CAR-T cells during killing of glioma cells *in vitro***

Feng Ji^1, 2^, Kexing Gao^1^, Fei Wang^2^, Jing Yu^1, 2^, Yan Shi^3^, Xianchen Wu^4*^, Hao Lin^1*^

*1 Office of Research Platform Management, Zhongda Hospital, School of Medicine, Southeast University, Nanjing, China, 210009.*

*2* *National Medical Research of Industry-Education Integration lnnovation Platform for Interventional Medica Engineering, Zhongda Hospital, Southeast University, Nanjing,* *China, 210031.*

*3* *Nanjing First Hospital, Nanjing, China, 210006.*

*4* *Shuyang Central Hospital, Suqian, China, 223600.*

**Running title:** RVG29 co-expression promotes CAR-T for gliomas.

**Correspondence:** Hao Lin, Office of Research Platform Management, Zhongda Hospital, School of Medicine, Southeast University, Nanjing, China, 210009. E-mail: [haolin@seu.edu.cn](mailto:haolin@seu.edu.cn); Xianchen Wu, Shuyang Central Hospital, Suqian, China. E-mail: 13485076338@163.com.

**Supplementary Figures**


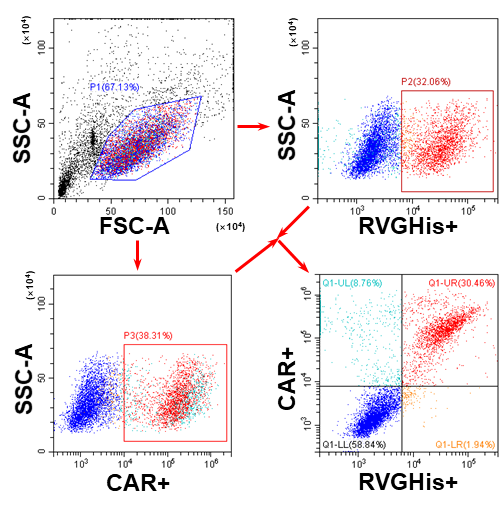


**Supplementary Figure S1. Gating Strategy for CAR-Positive Cell Detection.**


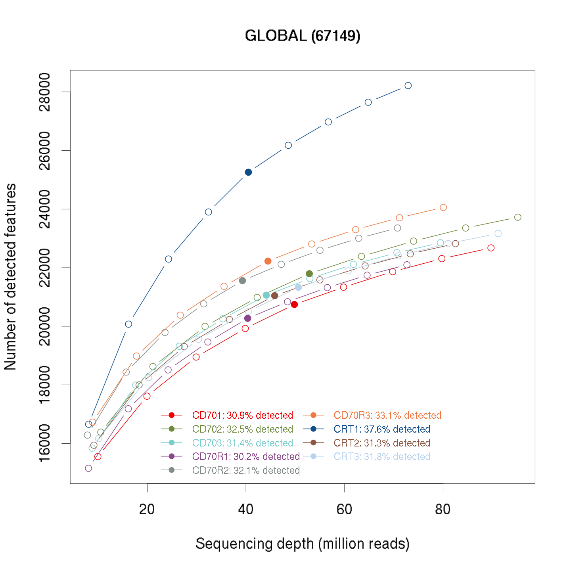


**Supplementary Figure S2. Saturation analysis of the transcriptome sequencing.**

The x-axis represents the randomly sampled sequencing data volume (unit: million reads), and the y-axis represents the number of genes detected at that data volume. As shown in the figure, the curve reaches a plateau when the sequencing depth reaches approximately 40 million reads, indicating that the current sequencing depth is sufficient to stably detect the vast majority of expressed genes in the samples, and the sequencing data volume is adequate and efficient.


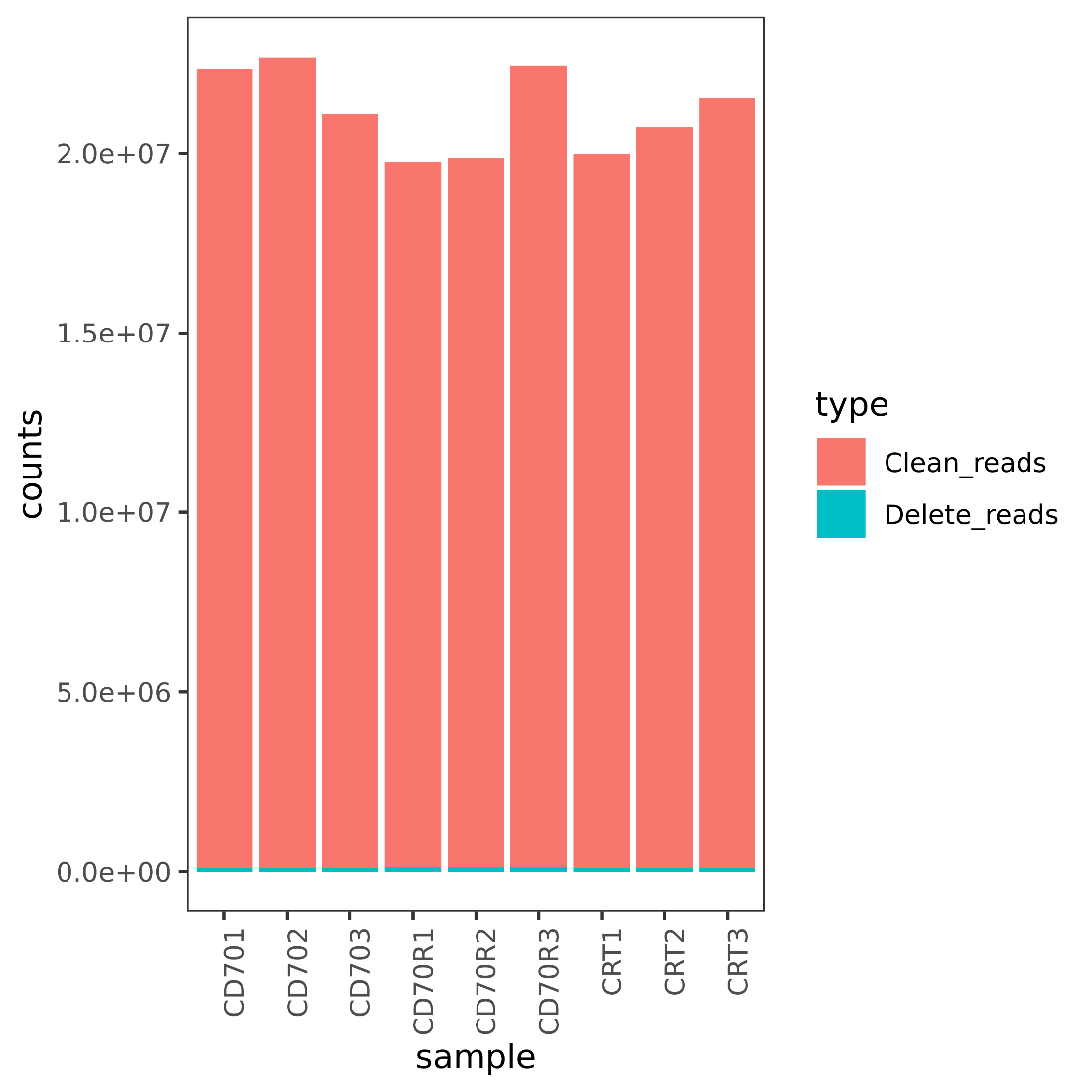


**Supplementary Figure S3. Statistics of sequencing data quality control and filtering.**

Raw sequencing data were processed using Trim Galore! to remove adapter sequences, low-quality bases, N bases, and sequences shorter than a length threshold (default: 20 bp). The bar chart shows the number of sequences before and after filtering for each sample, where blue represents high-quality sequences retained after QC, and gray represents the removed sequences. The high percentage of valid data in all samples indicates good quality of the raw data.


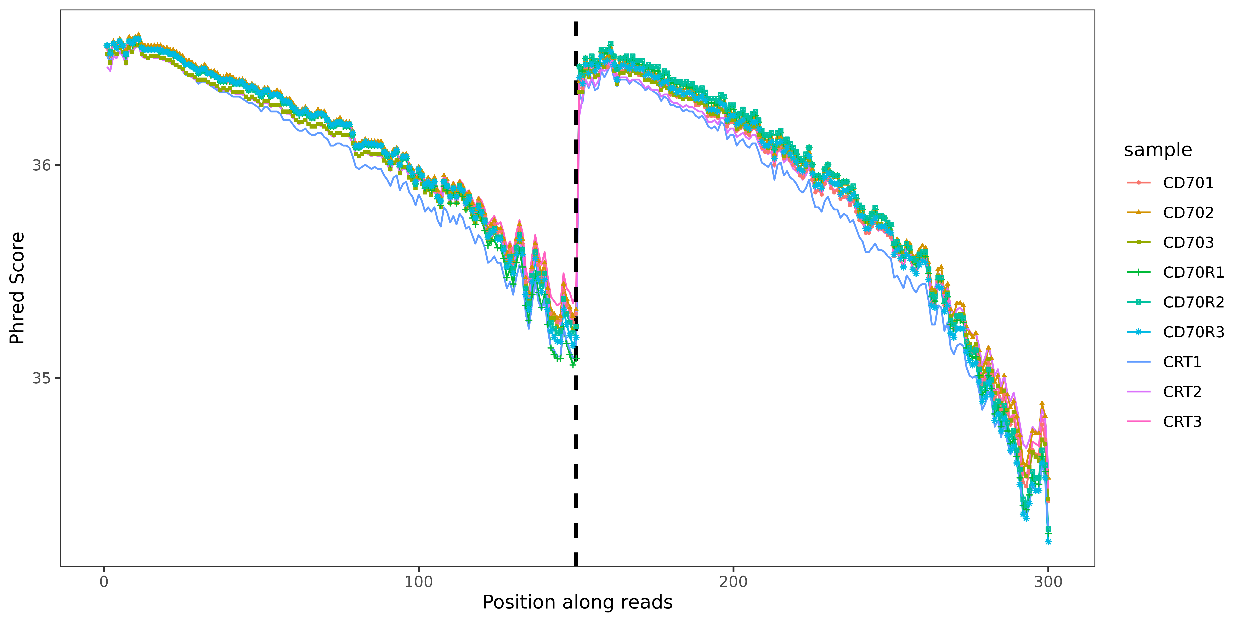


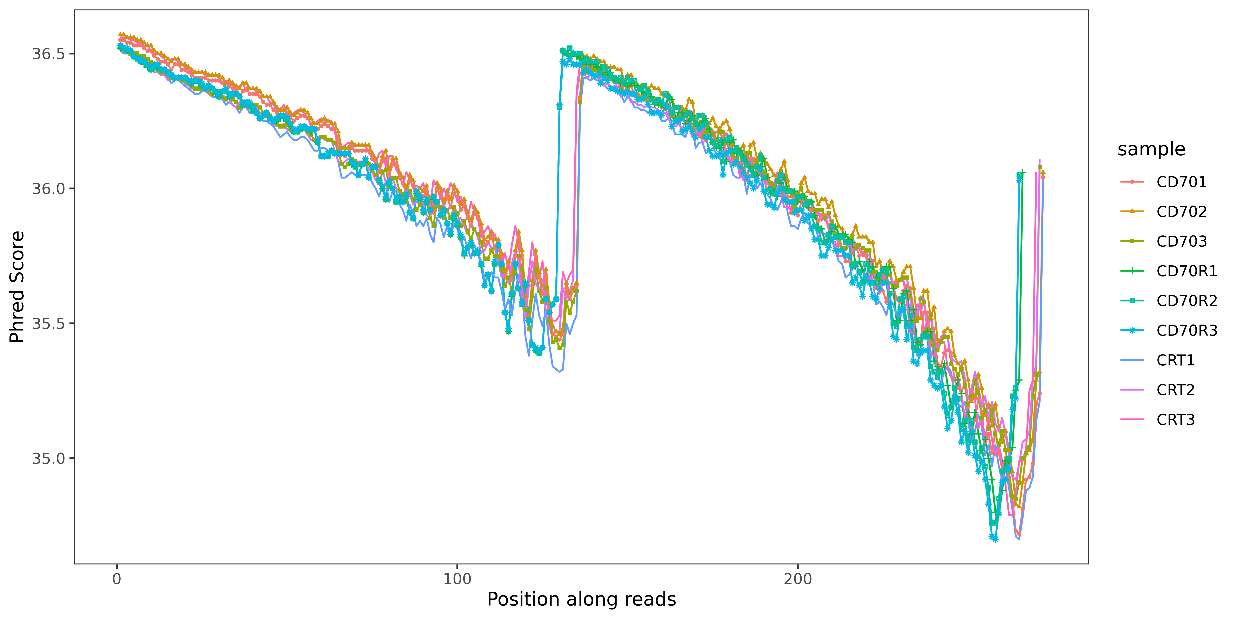


**Supplementary Figure S4. Base quality distribution of the sequencing data.**

The upper and lower panels show the average base quality distribution across all sequencing cycles for each sample before and after data quality control, respectively. The x-axis represents the position within the read, and the y-axis represents the Phred quality score (Q-score; Q = -10log₁₀(e)), where Q20 and Q30 represent error rates of 1% and 0.1%, respectively. The dashed line separates Read 1 and Read 2 of the paired-end sequencing. The overall base quality remains high both before and after QC, indicating a stable and reliable sequencing process.


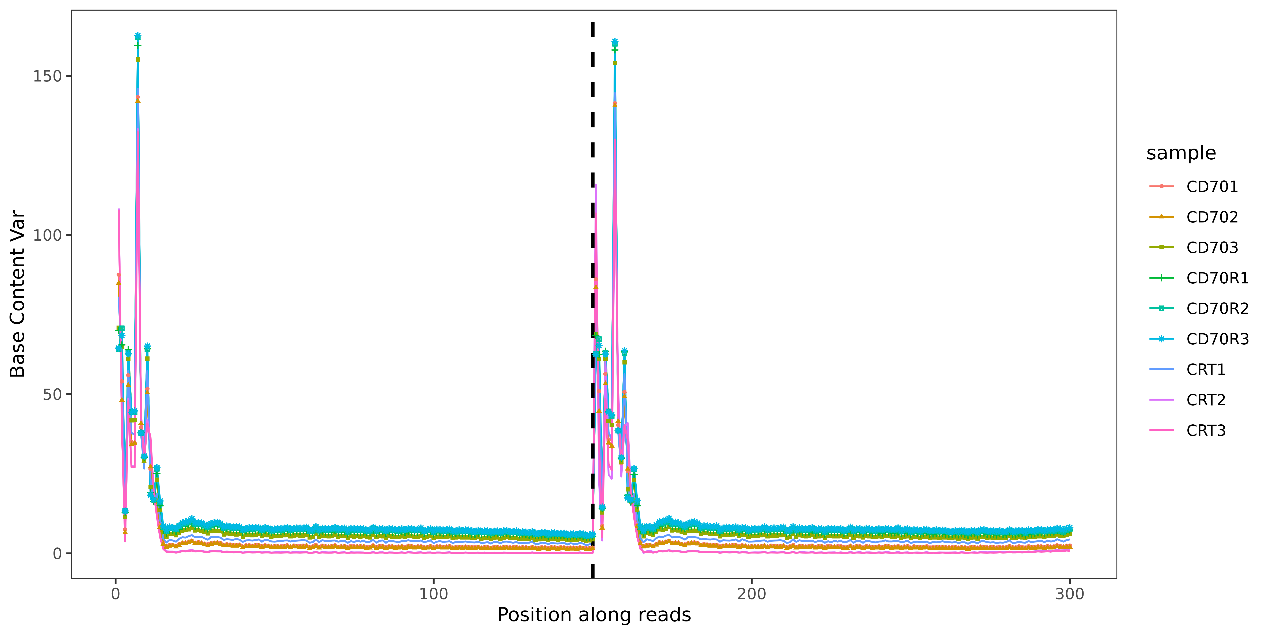


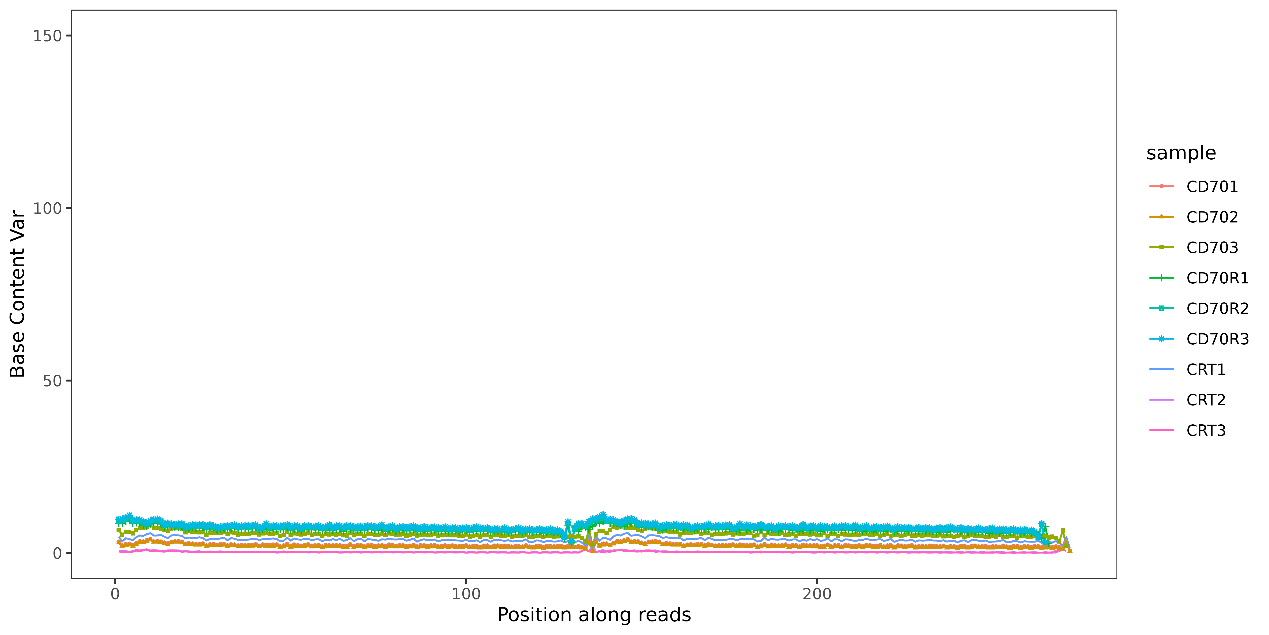


**Supplementary Figure S5. Nucleotide composition distribution of the sequencing data.**

The upper and lower panels show the variance in GC content distribution across all sequencing cycles for each sample before and after data quality control, respectively. The x-axis represents the position within the read, and the y-axis represents the variance of the GC content. After quality control filtering, the GC content distribution curves of all samples become more uniform and stable, indicating the absence of significant sequence-specific contamination and an overall improvement in data quality.

**
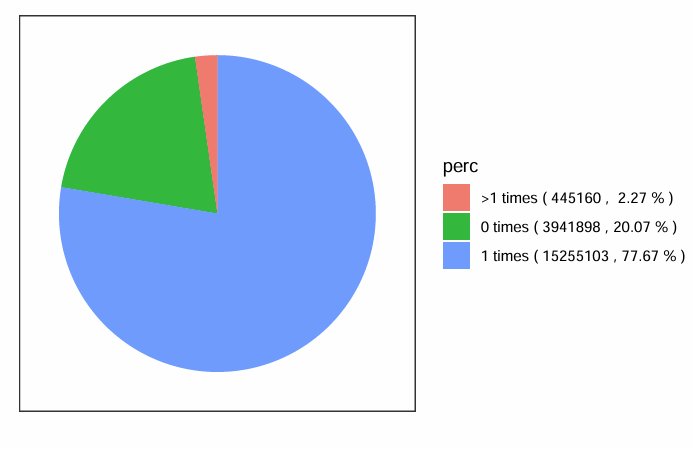

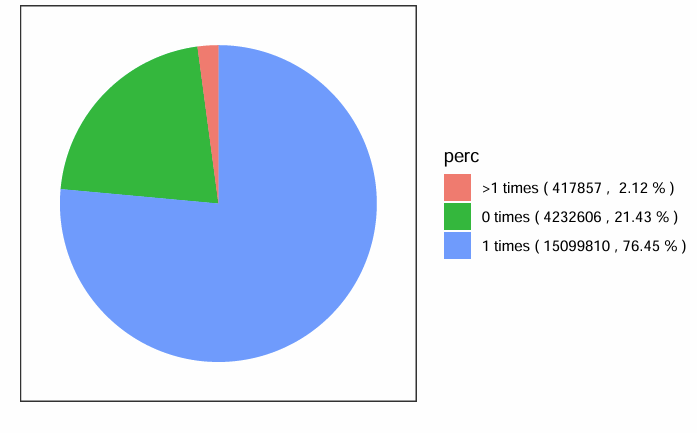

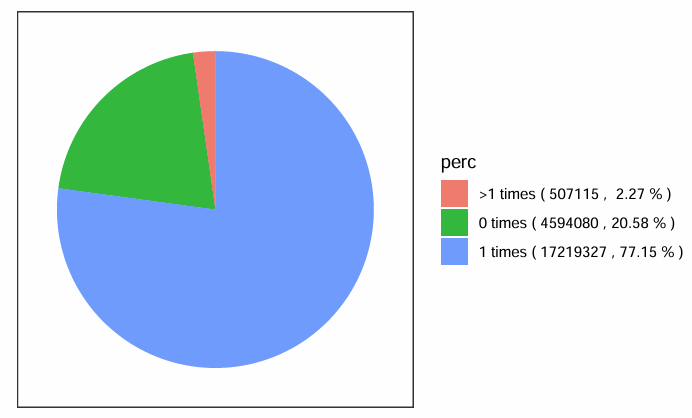
**

**
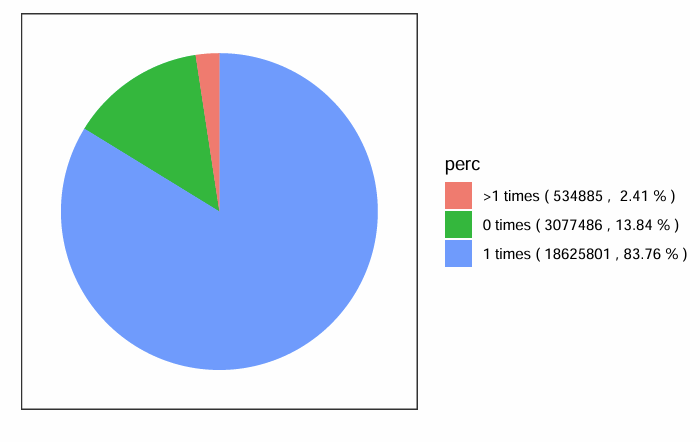

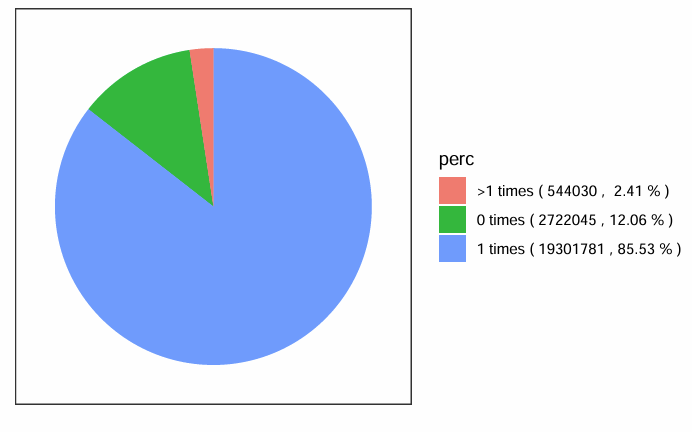

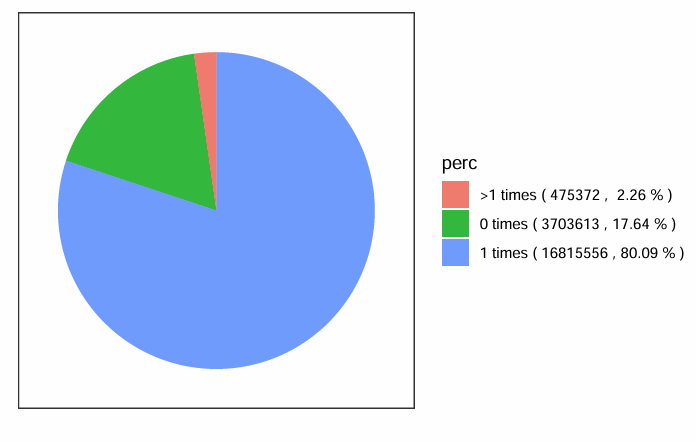

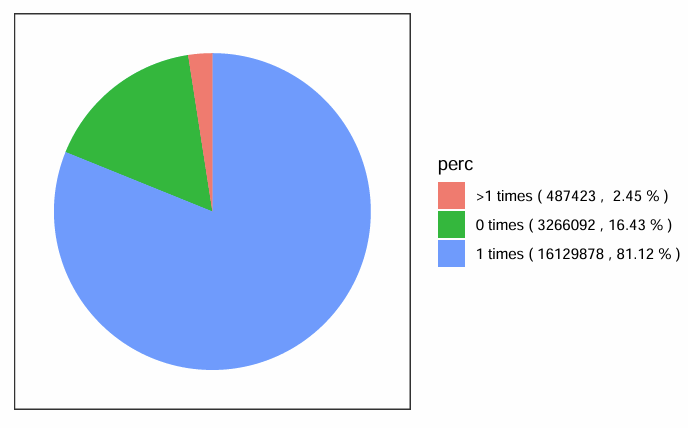

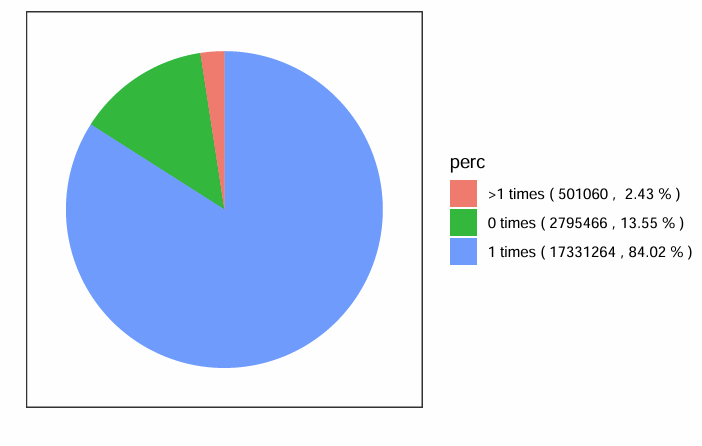

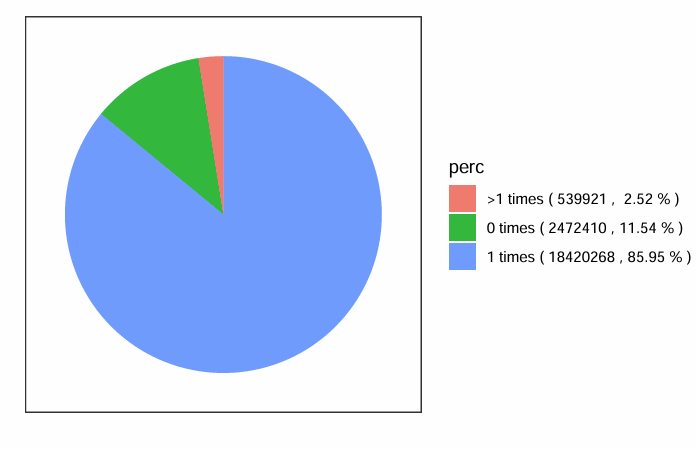
**

**Supplementary Figure S6. Statistics of sequencing read alignment efficiency to the reference genome.**

The bar chart shows the alignment efficiency of quality-controlled reads to the reference genome for each sample. Blue represents the proportion of reads that could be uniquely mapped to the genome, which were used for subsequent analysis; red and gray represent the proportions of multi-mapped reads and unmapped reads, respectively. The unique mapping rate for all samples was higher than 75%, indicating no significant contamination in the experiment and good compatibility between the reference genome and the sample species.


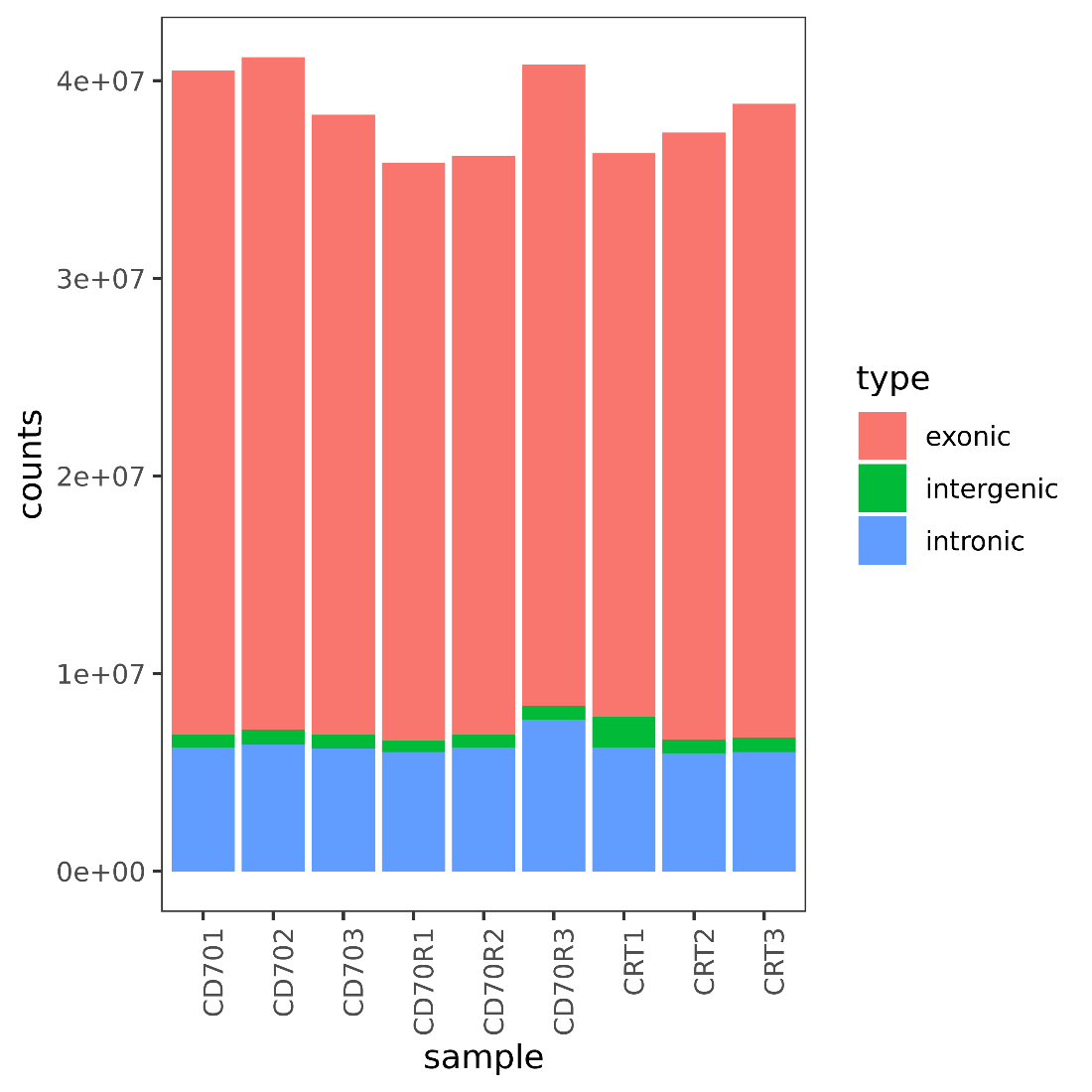


**Supplementary Figure S7. Distribution of uniquely mapped reads across genomic functional regions.**

The distribution proportions of uniquely mapped reads across exonic, intronic, and intergenic regions of the genome are shown. As illustrated, reads mapped to exonic regions (red) dominate in all samples. This pattern aligns with the expectation of a standard mRNA-Seq experiment, strongly demonstrating successful enrichment of mature mRNA, good RNA integrity, and the absence of significant genomic DNA contamination or RNA degradation.


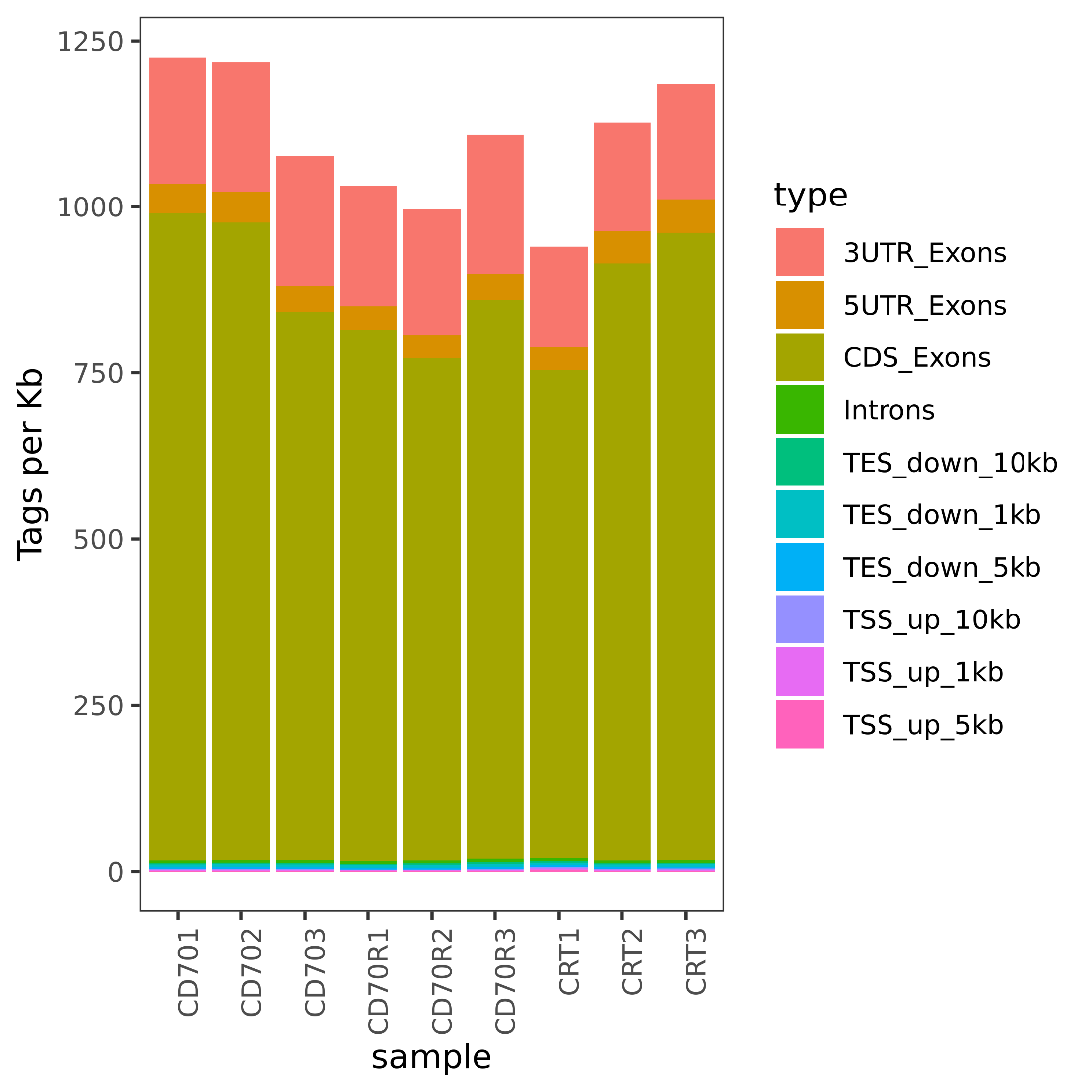


**Supplementary Figure S8. Analysis of read coverage density across genomic functional regions.**

To account for differences in region lengths, this plot uses the normalized metric Tags/Kb (number of tags per kilobase) to measure read coverage density in different functional regions. The Tags/Kb value in the exonic regions is significantly higher than in other regions for all samples, further confirming the efficient capture of target transcripts and providing a high-quality data foundation for subsequent gene expression quantification analysis.


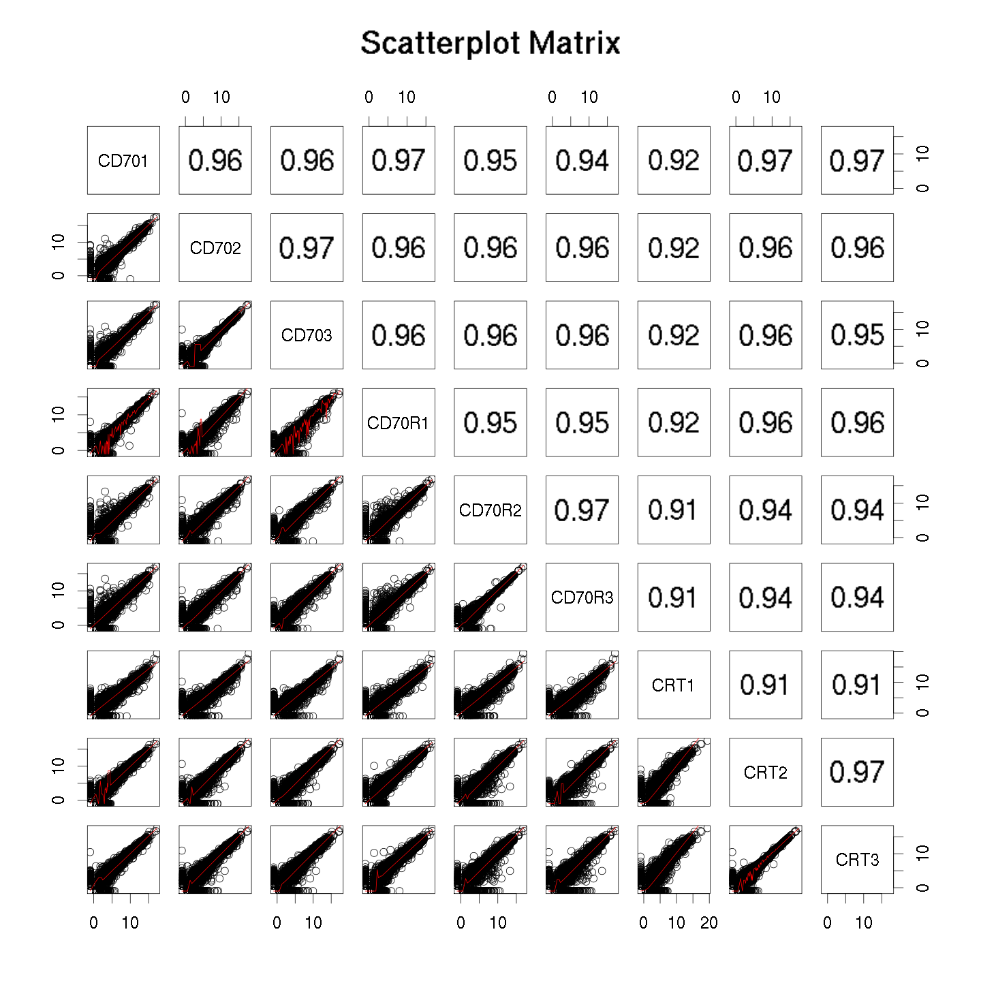


**Supplementary Figure S9. Scatter plot matrix of gene expression correlation between samples.**

This matrix displays the pairwise correlation of gene expression levels between all samples. The values shown are Pearson correlation coefficients (R²). The R² values for all samples, particularly between biological replicates within groups, are above 0.9, indicating high inter-sample correlation and excellent experimental reproducibility, meeting the statistical requirements for subsequent differential expression analysis.


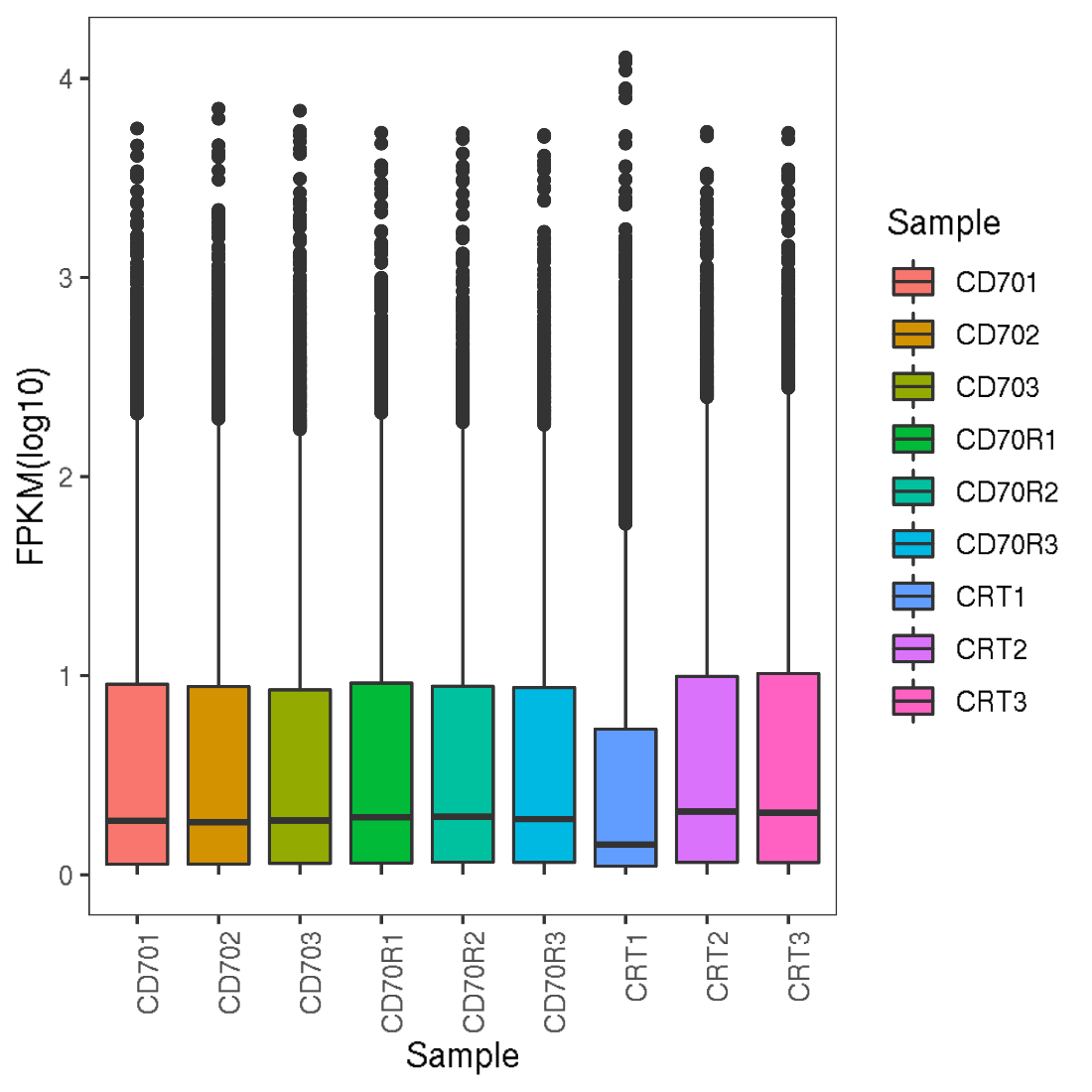


**Supplementary Figure S10. Boxplot of gene expression level distribution across samples.**

The boxplot displays the distribution of normalized gene expression levels (log₁₀(FPKM)) for all samples. The median, upper/lower quartiles, and dispersion range of expression levels are highly similar across all samples, indicating consistent sequencing depth, data normalization, and overall transcript abundance distribution, confirming stable and reliable data quality.


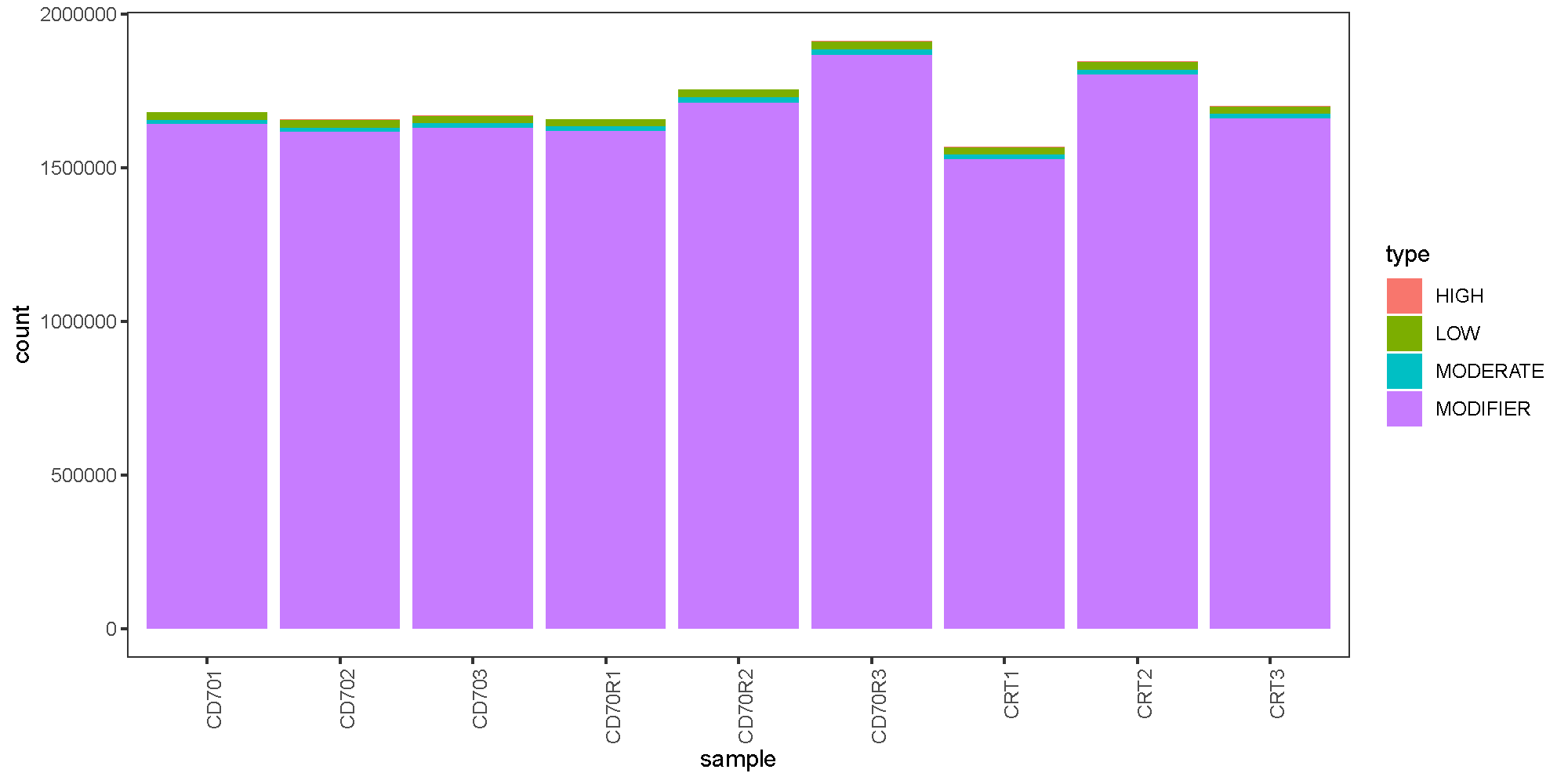


**Supplementary Figure S11. Classification statistics of functional impact of genetic variants.**

Based on SNPs and InDels detected by GATK, variants were categorized according to their potential functional impact on genes using SnpEff. The relative proportions of High, Moderate, Low, and Modifier impact variants show a consistent trend across all samples. This indicates that all samples share a similar genetic background and that no significant batch effects or technical biases were introduced throughout the experimental workflow and data analysis process.

**Supplementary Table 1**

| **Primer** | **Sequence (5’- 3’)** |
| --- | --- |
| HSH2D-F | TCCCGCAAACGTGGATTACG |
| HSH2D-R | GGCTTCCTTGGTGGTTATTCTG |
| TXNIP-F | GGTCTTTAACGACCCTGAAAAGG |
| TXNIP-R | ACACGAGTAACTTCACACACCT |
| SREBF1-F | ACAGTGACTTCCCTGGCCTAT |
| SREBF1-R | GCATGGACGGGTACATCTTCAA |
| ATG2A-F | GCTCAGGGTACATGGAGCTG |
| ATG2A-R | CTCGTGGTCTGTAAGGCTCAC |
| SFPQ-F | AGCGATGTCGGTTGTTTGTTG |
| SFPQ-R | AGCGAACTCGAAGCTGTCTAC |
| HBEGF-F | ATCGTGGGGCTTCTCATGTTT |
| HBEGF-R | TTAGTCATGCCCAACTTCACTTT |
| MYC-F | GGCTCCTGGCAAAAGGTCA |
| MYC-R | CTGCGTAGTTGTGCTGATGT |
| TAL1-F | AGCCGGATGCCTTCCCTAT |
| TAL1-R | GGGACCATCAGTAATCTCCATCT |
| CXCL9-F | CCAGTAGTGAGAAAGGGTCGC |
| CXCL9-R | AGGGCTTGGGGCAAATTGTT |
| TNFRSF18-F | ACCCAGTTCGGGTTTCTCAC |
| TNFRSF18-R | CCAGATGTGCAGTCCAAGC |
| LGALS12-F | AACCCTCGCTTCCATACCAC |
| LGALS12-R | TCCTCATTCCCGAAGAGAAAGAG |
| RORC-F | GTGGGGACAAGTCGTCTGG |
| RORC-R | AGTGCTGGCATCGGTTTCG |
| SOS1-F | GAGTGAATCTGCATGTCGGTT |
| SOS1-R | CTCTCATGTTTGGCTCCTACAC |
